# Granulocyte colony-stimulating factor protects against acute systemic alphavirus disease in a type I IFN-dependent manner

**DOI:** 10.3389/fimmu.2025.1606053

**Published:** 2025-07-11

**Authors:** Muddassar Hameed, Pallavi Rai, Md Shakhawat Hossain, Andrea Daamen, Peter E. Lipsky, James Weger-Lucarelli

**Affiliations:** ^1^ Department of Biomedical Sciences and Pathobiology, Virginia-Maryland College of Veterinary Medicine, Virginia Tech, Blacksburg, VA, United States; ^2^ Center for Zoonotic and Arthropod-borne Pathogens, Virginia Tech, Blacksburg, VA, United States; ^3^ Department of Pathology and Immunology, Alvin J. Siteman Cancer Center, Washington University School of Medicine, St. Louis, MO, United States; ^4^ AMPEL BioSolutions LLC and the RILITE Research Institute, Charlottesville, VA, United States

**Keywords:** arthritogenic alphaviruses, chikungunya virus, Mayaro virus, G-CSF, neutrophils

## Abstract

**Introduction:**

Arthritogenic alphaviruses, including chikungunya (CHIKV) and Mayaro virus (MAYV), cause disease characterized by fever, rash, and incapacitating joint pain. Alphavirus arthritis is associated with infiltration of myeloid cells and increases in several cytokines systemically, including granulocyte colony-stimulating factor (G-CSF). G-CSF is secreted by endothelial cells, fibroblasts, macrophages, and monocytes and binds to colony-stimulating factor 3 receptor (CSF3R, also known as G-CSFR) on the surface of myeloid cells. G-CSFR signaling initiates the proliferation, differentiation, and maturation of myeloid cells, especially neutrophils. Importantly, G-CSF has been found at high levels in both the acute and chronic phases of chikungunya disease; however, the role of G-CSF in arthritogenic alphavirus disease remains unexplored.

**Methods:**

Here, we sought to test the effect of G-CSF on CHIKV and MAYV infection using G-CSFR-deficient mice (G-CSFR^-/-^).

**Results:**

Compared to wild-type mice, we observed sustained weight loss in G-CSFR^-/-^ mice following CHIKV and MAYV infection. Furthermore, G-CSFR^-/-^ mice had a significantly higher percentage of inflammatory monocytes and a reduction in neutrophils throughout infection. The difference in weight loss in G-CSFR^-/-^ mice induced by alphavirus infection was corrected by blocking type I IFN signaling.

**Discussion:**

In summary, these studies suggest that type I IFN signaling contributes to G-CSFR-mediated control of arthritogenic alphavirus disease. Therefore, G-CSF or G-CSFR may be therapeutic targets to modulate host immune responses against arthritogenic alphavirus disease.

## Introduction

Arthritogenic alphaviruses, including chikungunya (CHIKV), Ross River (RRV), O’nyong nyong (ONNV), and Mayaro virus (MAYV) are significant public health threats (1, 2). These viruses are spread worldwide: CHIKV is endemic to Africa, Southeast Asia, and, more recently, the Caribbean and South America.; RRV, ONNV, and MAYV are endemic to Australia, Africa, and South America, respectively (3). The disease caused by arthritogenic alphaviruses is characterized by high fever, rash, myositis, and arthritis, which can persist for years in roughly half of affected patients (4–9). However, there are no specific therapies available for the treatment of alphavirus arthritis except the use of anti-inflammatory drugs for symptomatic relief (10, 11). Thus, there is an urgent need to understand the immune mechanisms that control arthritogenic alphavirus disease outcomes in order to develop therapeutics.

During arthritogenic alphavirus infection, the virus replicates in fibroblasts, muscle satellite cells, macrophages, and other cells, initiating inflammatory responses (12, 13). This results in the influx of various immune cells, including myeloid cells, leading to tissue damage and the production of proinflammatory cytokines, including granulocyte colony-stimulating factor (G-CSF) (14–16). G-CSF is secreted by several cell types, including endothelial cells, fibroblasts, macrophages, monocytes, and bone marrow stromal cells (reviewed in (17, 18)). Its function is driven by binding to its cognate receptor, the G-CSF receptor (G-CSFR) (19–21), on the surface of myeloid precursor cells and some non-immune cells. Binding initiates signal transduction and activation of cellular pathways that drive proliferation, differentiation, and maturation of granulocytes, especially neutrophils (22, 23), which play an important role in arthritogenic alphavirus pathogenesis (24–26). In addition to its role in regulating granulopoiesis, G-CSF has been implicated in host responses to various viral infections. Elevated G-CSF levels have been observed in patients infected with influenza A virus and SARS-CoV-2, where it is associated with disease severity and neutrophil recruitment (22–24). These findings suggest that G-CSF may contribute to both protective and pathogenic immune responses during viral infections. However, its specific role in the context of arthritogenic alphavirus infections remains poorly understood. Previous studies have shown that G-CSF is upregulated in acute and chronic arthritogenic alphavirus-infected humans (25–28) and mice (29), and that higher influx of neutrophils into joint tissues during CHIKV infection is associated with worse disease outcomes in mice (20, 30). Furthermore, MyD88-dependent influx of neutrophils and monocytes impairs lymph node B cell responses to CHIKV infection (24). Collectively, these data highlight the important role of G-CSF and neutrophils in arthritogenic alphavirus infection, which is still vastly understudied.

In this study, we investigated the contribution of G-CSF signaling in the development of CHIKV and MAYV-induced disease. We infected G-CSFR knockout (G-CSFR^-/-^) (23) mice with CHIKV and MAYV and monitored disease development. We observed a sustained weight loss in infected G-CSFR^-/-^ animals compared to wild-type (WT) control groups. Blood immune cell profiling showed that G-CSFR deficiency led to decreased neutrophils and a significant increase in monocytes throughout infection. Alphaviruses are highly sensitive to type I IFN restriction, and monocytes are known to produce type I IFNs in response to CHIKV or RRV infection (31, 32). Therefore, we blocked IFN signaling, which corrected the weight loss differences observed between G-CSFR^-/-^ and WT mice. Future studies can be conducted to further dissect the role of G-CSF in mediating inflammatory responses during arthritogenic alphavirus infection and the potential of targeting G-CSF or its receptor therapeutically.

## Materials and methods

### Ethics statement

All experiments were conducted with the approval of Virginia Tech’s Institutional Animal Care & Use Committee (IACUC) under protocol number 24-060. Experiments using CHIKV and MAYV were performed in a BSL-3 or BSL-2 facility, respectively, in compliance with CDC and NIH guidelines and with approval from the Institutional Biosafety Committee (IBC) at Virginia Tech.

### Mice

C57BL/6J mice (strain# 000664) and colony-stimulating factor 3 receptor gene knockout mice (B6.129X1 (Cg)-Csf3r^tm1Link/J^; herein referred to as G-CSFR^-/-^) (23) were purchased from The Jackson Laboratory at 6–8 weeks of age. Heterozygous G-CSFR^+/-^ mice were bred at Virginia Tech to obtain homozygous G-CSFR^-/-^ mice. Representative genotyping images and the primers used to confirm homozygous colonies are presented in **Supplementary Figure S1**. Homozygous G-CSFR^-/-^ mice were used in all studies. Mice were kept in groups of four or five animals per cage at ambient temperature with *ad libitum* supply of food and water.

### Cell culture and viruses

Vero cells were obtained from the American Type Culture Collection (ATCC; Manassas, VA) and grown in Dulbecco’s Modified Eagle’s Medium (DMEM, Gibco) with 5% fetal bovine serum (FBS, Genesee), 1 mg/mL gentamicin (Thermo Fisher), 1% non-essential amino acids (NEAA, Sigma) and 25 mM HEPES buffer (Genesee) at 37°C with 5% CO_2_. CHIKV SL-15649 (ECSA lineage), rescued from an infectious clone, was a gift from Dr. Mark Heise (the University of North Carolina at Chapel Hill (33), and MAYV strain TRVL 4675 was derived from an infectious clone (34, 35). Virus titers were determined by plaque assay on Vero cells, as previously described (36). The limit of detection for plaque assays is defined as one plaque-forming unit at the lowest dilution tested; samples below the limit of detection were assigned an arbitrary value of nine plaques, one dilution below what was tested, which was typically an undiluted sample.

### Mouse infections

Mice were injected in both hind footpads with 10^2^ PFU or 10^5^ PFU of CHIKV SL-15649 or 10^4^ PFU of MAYV in 50 μL viral diluent in each foot (37, 38). All virus dilutions were made in RPMI-1640 media with 10 mM HEPES and 2% FBS, hereafter referred to as viral diluent. Mice were monitored for disease development following infection through daily weighing, and footpad swelling was measured using a digital caliper. Blood was collected via submandibular bleed for serum isolation to determine viremia and cytokine levels. The limit of detection (LOD) for plaque assays was defined as one plaque-forming unit at the lowest dilution tested. Samples below the limit of detection were assigned an arbitrary value of nine plaques, corresponding to one dilution below the tested range, which was typically an undiluted sample. At sixteen or twenty-one-days post-infection (dpi), mice were euthanized, and blood was collected to isolate immune cells.

### Luminex assay and ELISA

We quantified G-CSF levels in the serum of mock- and CHIKV/MAYV-infected animals using the mouse Luminex XL cytokine assay (Bio-Techne) and the G-CSF DuoSet ELISA kit (Catalog# DY414-05, R&D Systems) according to the manufacturer’s instructions. Serum IFNγ and TNFα levels were measured using DuoSet ELISA kits, also from R&D Systems. A standard curve was generated using the optical density values of the standards, which were used to estimate the cytokine levels in each sample.

### Type I IFN signaling blockage

For *in vivo* interferon-α/β receptor (IFNAR) blockade, C57BL/6J and G-CSFR^-/-^ mice were intraperitoneally (i.p.) inoculated with 0.1 mg of MAR1–5A3, anti-IFNAR antibody (Leinco Technologies, Fenton, MO) (39) one day prior to infection. Mice were then inoculated with 10^2^ PFU of CHIKV SL-15649 in 50 μL of viral diluent in both hind feet and monitored for disease development until 21 dpi.

### Mouse blood immune cell isolation

Mouse blood leukocytes were isolated using Mono-Poly resolving medium (M-P M, MP Bio, Cat. No. 091698049) according to the manufacturer’s instructions. Briefly, blood was mixed with an equal volume of PBS and layered slowly onto M-P M followed by centrifugation at 300 × *g* for 30 min in a swinging bucket rotor at room temperature (20–25°C). We collected cell layers between the plasma and M-P M to isolate leukocytes and added them to a 15 mL conical tube containing 10 mL of cold 10% FBS containing RPMI-1640 (RPMI-10). Cells were spun at 500 × *g* for 5 min at 4°C and used for flow cytometry.

### Flow cytometry

Single cell suspensions were washed with PBS and resuspended in 100 μL Zombie aqua cell viability dye solution (1:400 prepared in PBS, Cat. No. 423101, BioLegend) and incubated at room temperature for 15–30 minutes. 200 μL flow cytometry staining (FACS) buffer (PBS containing 2% FBS) was added and centrifuged at 500 × *g* for 5 min at 4°C. The resulting cell pellet was resuspended in FACS buffer with 0.5 mg/mL rat anti-mouse CD16/CD32 Fc block (Cat. No. 553142, BD Biosciences) and incubated for 15 min on ice to block Fc receptors. For extracellular staining, a combined antibody solution was prepared in FACS buffer with fluorophore-conjugated antibodies: anti-mouse Alexa fluor 700 CD45 (Cat. No. 103128, BioLegend), anti-mouse PerCP/Cyanine 5.5 CD11b (Cat. No. 101227, BioLegend), anti-mouse APC Ly6G (Cat. No. 127614, BioLegend), anti-mouse PE Ly6C (Cat. No. 128007, BioLegend), anti-mouse PE CD3 (Cat. No. 100206, BioLegend), anti-mouse PerCP/Cyanine 5.5 CD4 (Cat. No. 116012, BioLegend), and anti-mouse FITC CD8a (Cat. No. 100706, BioLegend), and APC CD19 (Cat. No. 152410, BioLegend). 100 μL antibody cocktail was added to the single cell suspension, mixed, and incubated for 30 min on ice. Cells were washed with FACS buffer twice, and 100 μL 4% formalin (Thermo Fisher Scientific, Ref. No. 28908) was added to fix the cells. After 15 min incubation at room temperature, cells were washed with FACS buffer, resuspended in 100-200 μL PBS, and covered with aluminum foil before flow cytometry analysis. For each antibody, single color controls were run with Ultracomp ebeads (Cat. No. 01-2222-42, Thermo Fisher Scientific). The stained cells were analyzed using the FACSAria Fusion Flow cytometer (BD Biosciences).

### Statistical analysis

Statistical comparisons were performed using GraphPad Prism version 10. Data are presented as mean ± standard deviation. The statistical tests used to analyze data are described in figure legends.

## Results

### 1.1 G-CSF is upregulated in response to CHIKV and MAYV infection in C57BL/6J mice

**Figure 1:**
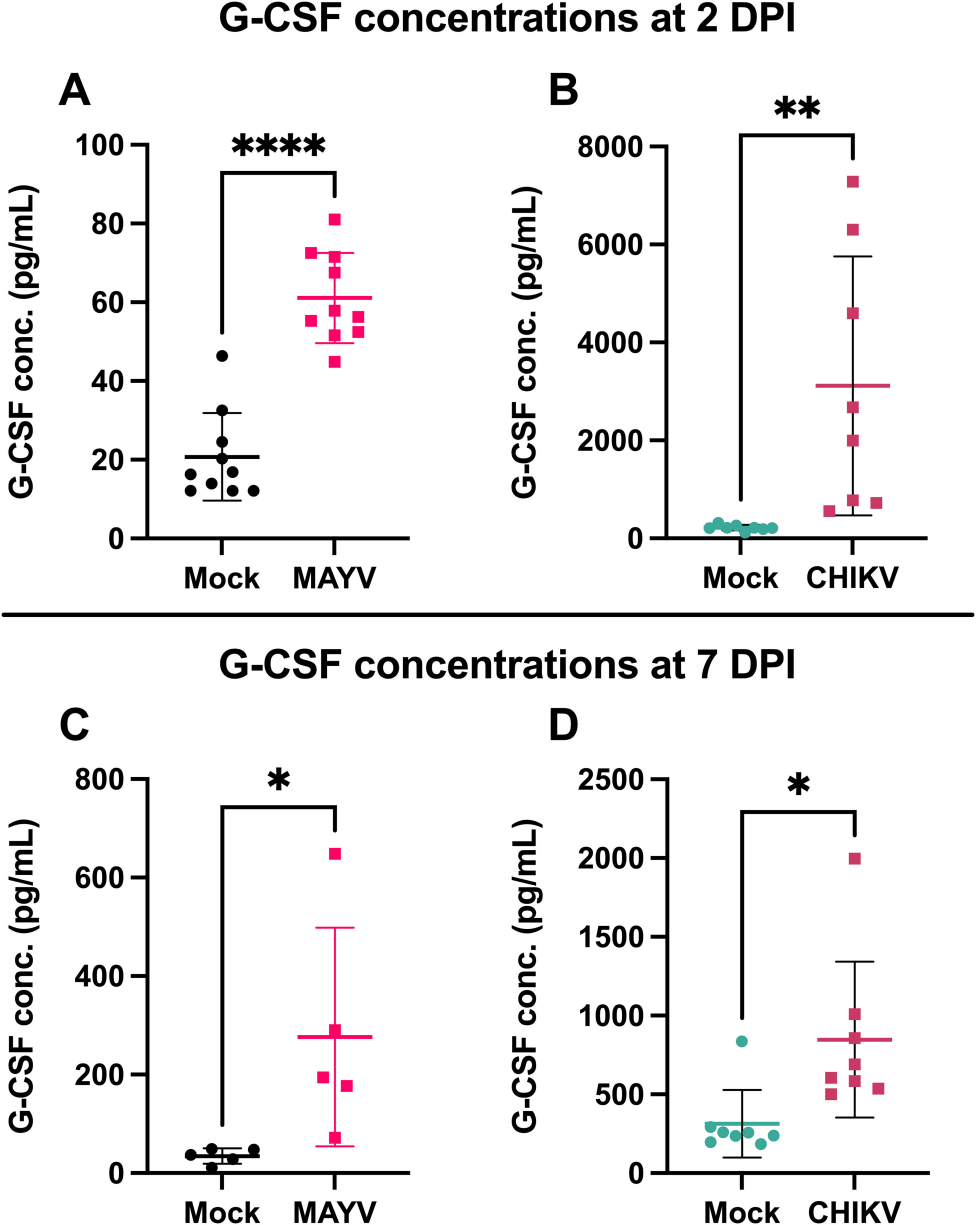
G-CSF is upregulated in response to CHIKV and MAYV infection in C57BL/6J mice. 6–8-week-old C57BL/6J mice were inoculated with viral diluent (mock), 10^5^ PFU of CHIKV strain SL-15649, or 10^4^ PFU of MAYV strain TRVL 4675 in each hind footpad, and blood was collected at 2 and 7 days post-infection (dpi) for serum isolation. **(A)** G-CSF levels were measured by Luminex assay at 2 dpi for MAYV. **(B-D)** G-CSF levels were measured by ELISA at 2 dpi for CHIKV **(B)** and 7 dpi for both MAYV **(C)** and CHIKV **(D)**. Values are expressed in picograms per milliliter of serum. The error bars represent the standard deviation, horizontal bars indicate mean values, and asterisks indicate statistical differences. Statistical analysis was done using an unpaired t-test; *p < 0.05; **p < 0.01; ****p < 0.0001. n = 10 for **(A)**, n = 8 for **(B, D),** and n = 5 for **(C)**.

G-CSF is elevated during arthritogenic alphavirus infection in humans (25–28) and mice (29). To validate G-CSF levels following arthritogenic alphavirus infection, we infected mice with CHIKV and MAYV and collected serum at 2 and 7 dpi, the peak of viremia and footpad swelling, respectively (37). At 2 dpi, G-CSF levels increased 3-fold (*p < 0.0001*) following MAYV infection (**Figure 1A**) and 14-fold following CHIKV (*p = 0.0079*; **Figure 1B**) compared to mock-inoculated controls. G-CSF levels remained higher at 7 dpi in MAYV (*p=0.04*) and CHIKV (*p=0.01*) infected mice compared to mock-infected groups (**Figures 1C, D**). These data validate that G-CSF levels increase following MAYV or CHIKV infection in mice.

### 1.2 Lack of G-CSF signaling leads to sustained weight loss in mice infected with arthritogenic alphaviruses

**Figure 2:**
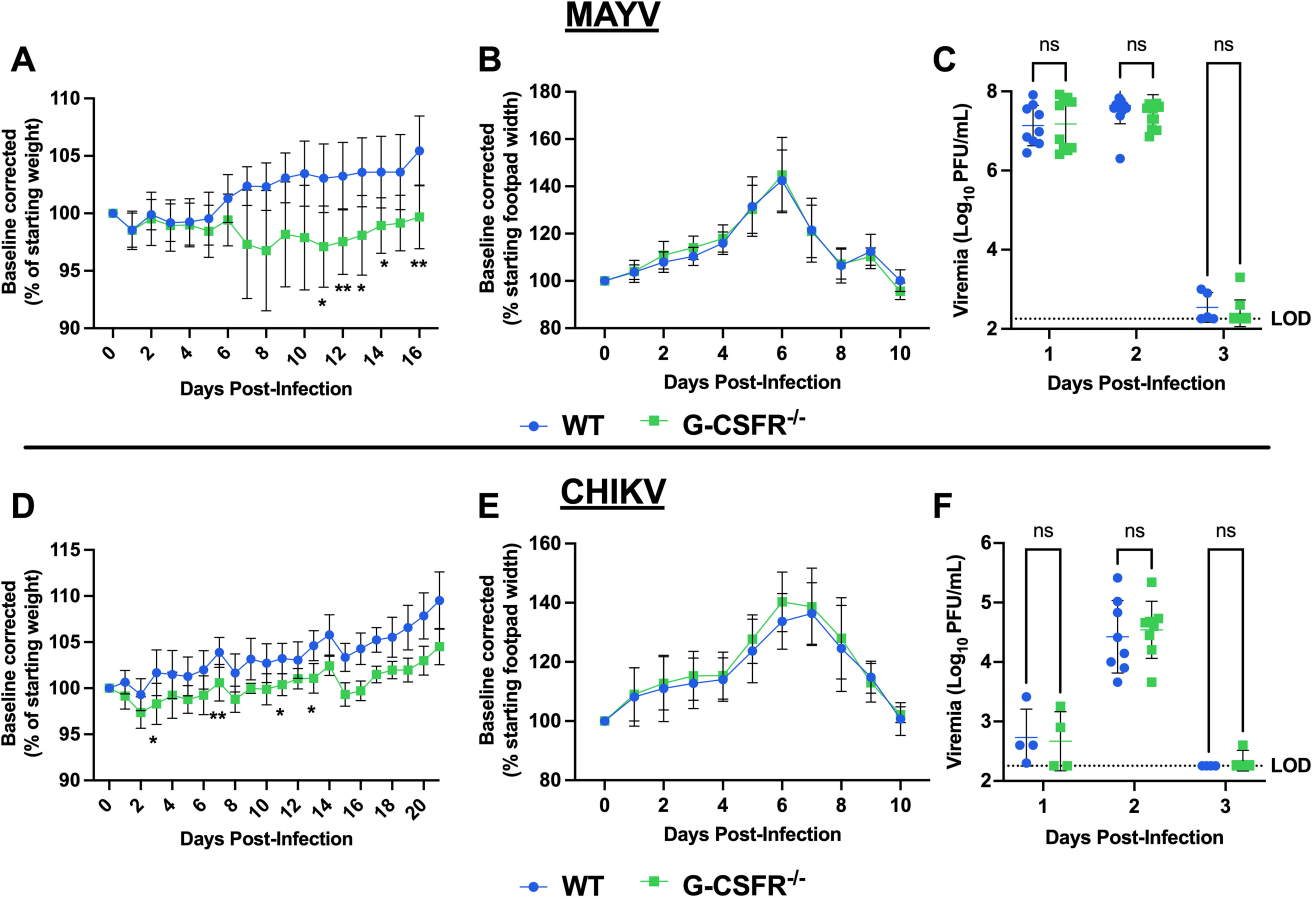
G-CSFR-deficient mice have sustained weight loss following CHIKV or MAYV infection. C57BL/6J and G-CSFR^-/-^ mice were inoculated with 10^4^ PFU of MAYV strain TRVL 4675 (n=10) or 10^5^ PFU of CHIKV strain SL-15649 (n=8) in each hind footpad and monitored for disease development until 16 and 21 dpi, respectively. **(A-C)** Weight loss **(A)**, footpad swelling **(B)**, and viremia **(C)** were assessed after MAYV infection. **(D-F)** Weight loss **(D)**, footpad swelling **(E)**, and viremia **(F)** were assessed after CHIKV infection. **(A-D)** Weight loss data are presented as a percentage of the starting weight of the mice on the day of inoculation. The baseline was given a value of 100%, and each mouse was tracked individually. Statistical analysis: Weight loss and footpad swelling: Mixed-effects analysis with Šídák’s multiple comparisons test. Viremia: Mixed-effects analysis or 2way ANOVA with Šídák’s multiple comparisons test. The error bars represent the standard deviation, bars indicate mean values **(C, F)**, the dotted line represents the limit of detection (LOD), and asterisks indicate statistical differences. The LOD was set to 1 plaque-forming unit (PFU) at the -1 dilution (200 PFU/mL); any negative sample was assigned an arbitrary value of 9 plaques in an undiluted sample (180 PFU/mL). The level of significance represented is as follows: *p < 0.05, **p < 0.01; ns, not significant (p < 0.05). Experiments were performed in two independent biological replicates with n = 8-12, except for panel **(F)** at 1 and 3 dpi, where n = 4.

G-CSFR is present on the surface of granulocyte precursors, initiating signal transduction and activation of cellular pathways that drive proliferation, differentiation, and maturation of granulocytes, especially neutrophils (22, 23). To assess the impact of G-CSF signaling on arthritogenic alphavirus disease outcomes, we inoculated mice genetically lacking the G-CSF receptor (G-CSFR^-/-^) with MAYV or CHIKV. Following MAYV infection, we observed sustained weight loss in G-CSFR^-/-^ mice compared to WT mice until 16 dpi (**Figure 2A**). No differences were observed in footpad swelling (**Figure 2B**) and virus replication (**Figure 2C**). CHIKV-infected G-CSFR^-/-^ mice showed a similar sustained weight loss compared to the WT group until 21 dpi (**Figure 2D**). As with MAYV, no differences were observed in footpad swelling (**Figure 2E**) and virus replication (**Figure 2F**) between the two groups. These data suggest that G-CSFR plays a role in recovery from systemic arthritogenic alphavirus disease.

### 1.3 Lack of G-CSF signaling leads to the systemic reduction of neutrophils and increase in monocytes during arthritogenic alphavirus infection

**Figure 3:**
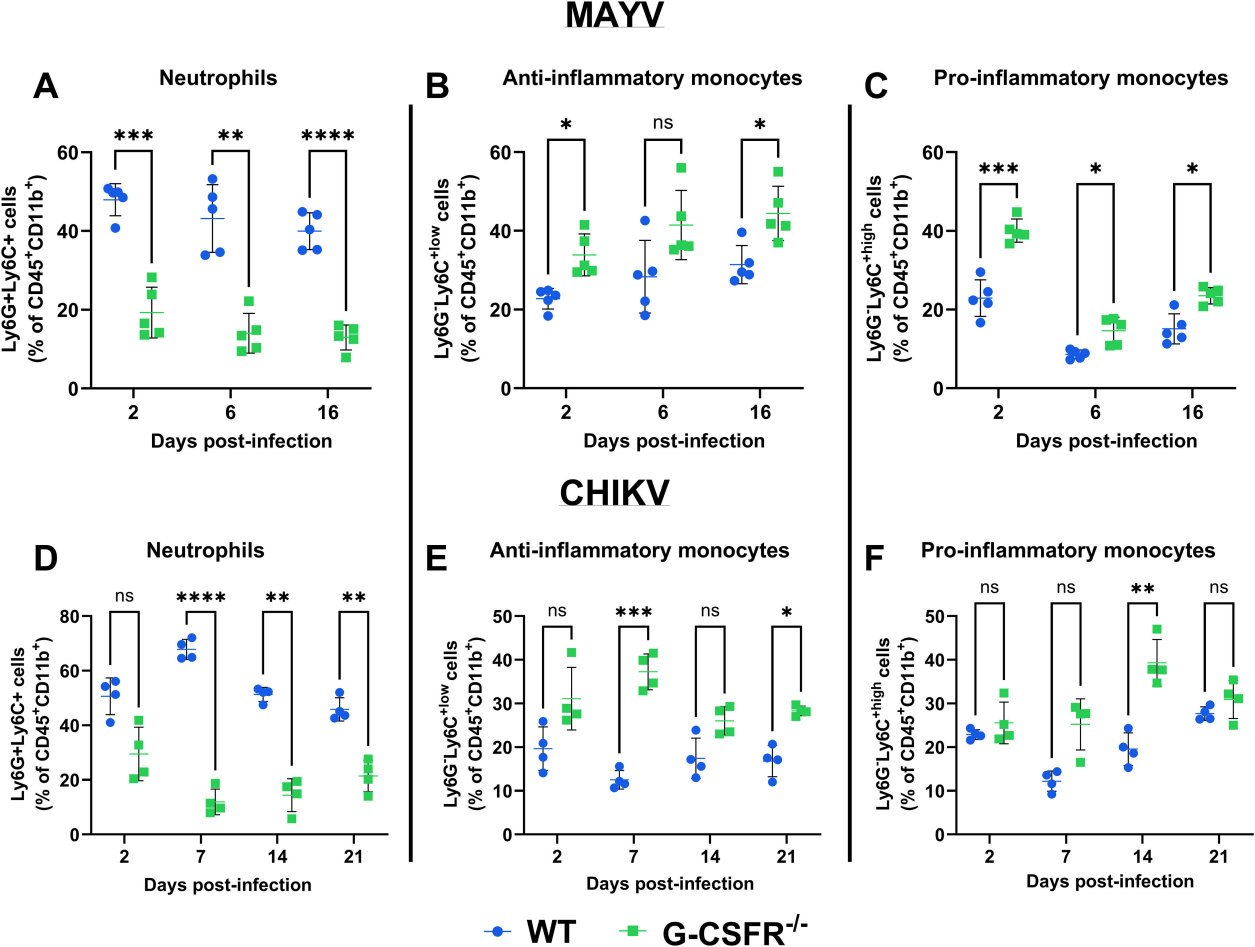
G-CSF deficiency leads to a systemic reduction in neutrophils and an increase in monocytes during arthritogenic alphavirus infection. C57BL/6J and G-CSFR^-/-^ mice were inoculated with 10^4^ PFU of MAYV strain TRVL 4675 (n=5) or 10^5^ PFU of CHIKV strain SL-15649 (n=4) in each hind footpad, and blood was collected at the indicated timepoints. Leukocytes were isolated from the blood and subjected to flow cytometry. **(A-F)** Dot plots presenting the percentage of neutrophils (CD45^+^CD11b^+^Ly6G^+^Ly6C^+^) **(A, D)**, anti-inflammatory monocytes (CD45^+^CD11b^+^Ly6G^-^Ly6C^lo^) **(B, E)**, and pro-inflammatory monocytes (CD45^+^CD11b^+^Ly6G^-^Ly6C^hi^) **(C, F)** in WT and G-CSFR^-/-^ groups during MAYV and CHIKV infection. Statistical analysis: 2way ANOVA with Šídák’s multiple comparisons test. The error bars represent the standard deviation, bars indicate mean values, and asterisks indicate statistical differences. The level of significance represented is as follows: *p < 0.05, **p < 0.01, ***p < 0.001, ****p <0.0001; ns, not significant (p < 0.05).

Next, we monitored the impact of the G-CSFR deficiency on the immune cell profile throughout infection. We isolated blood leukocytes at various time points following CHIKV and MAYV infection and profiled immune cell populations using flow cytometry (gating strategy presented in **Supplementary Figure S2**). As expected, G-CSFR deficiency resulted in a significant reduction in the proportion of neutrophils in the blood compared to wild-type (WT) mice during MAYV and CHIKV infection (**Figures 3A, D**). We also observed a significantly higher percentage of anti-inflammatory monocytes (Ly6C^lo^) in the blood of MAYV-infected G-CSFR^-/-^ mice at 16 dpi (**Figure 3B**) and CHIKV-infected G-CSFR^-/-^ mice at 7 and 21 dpi (**Figure 3E**) compared to WT controls. Pro-inflammatory monocytes (Ly6C^hi^) were higher in MAYV-infected G-CSFR^-/-^ mice at all time points tested (**Figure 3C**) and at 14 dpi in CHIKV-infected G-CSFR^-/-^ mice (**Figure 3F**); these cells trended higher in CHIKV-infected G-CSFR^-/-^ mice at 7 dpi, but the difference did not reach significance (*p = 0.059*). These data demonstrate that G-CSFR plays an important role in regulating myeloid populations in the blood during arthritogenic alphavirus infection.

We also determined the effect of G-CSFR^-/-^ deficiency on B and T cell dynamics during arthritogenic alphavirus infection. We observed no impact on CD4 and CD8 T cells in MAYV- and CHIKV-infected animals (**Supplementary Figures S3A, B, D, E**). In contrast, B cells were higher at 6 dpi in MAYV-infected G-CSFR^-/-^ mice and at 7 and 21 dpi in CHIKV-infected G-CSFR^-/-^ mice (**Supplementary Figures S3C, F**). To assess whether differences in B cell levels altered virus-specific antibody responses, we measured neutralization of MAYV at 13 dpi using a plaque reduction neutralization test with an 80% threshold (PRNT_80_). No differences were observed between the groups (**Supplementary Figure S4**). We also measured serum levels of IFNγ and TNFα at 6 dpi in MAYV-infected animals and observed no differences (**Supplementary Figure S5**). These data demonstrate that G-CSFR^-/-^ deficiency has minimal impacts on the proportion of T cells during infection but leads to a significant increase in B cells following infection with both viruses.

### 1.4 The impact of G-CSF on disease recovery depends on type I interferon signaling during arthritogenic alphavirus infection

**Figure 4:**
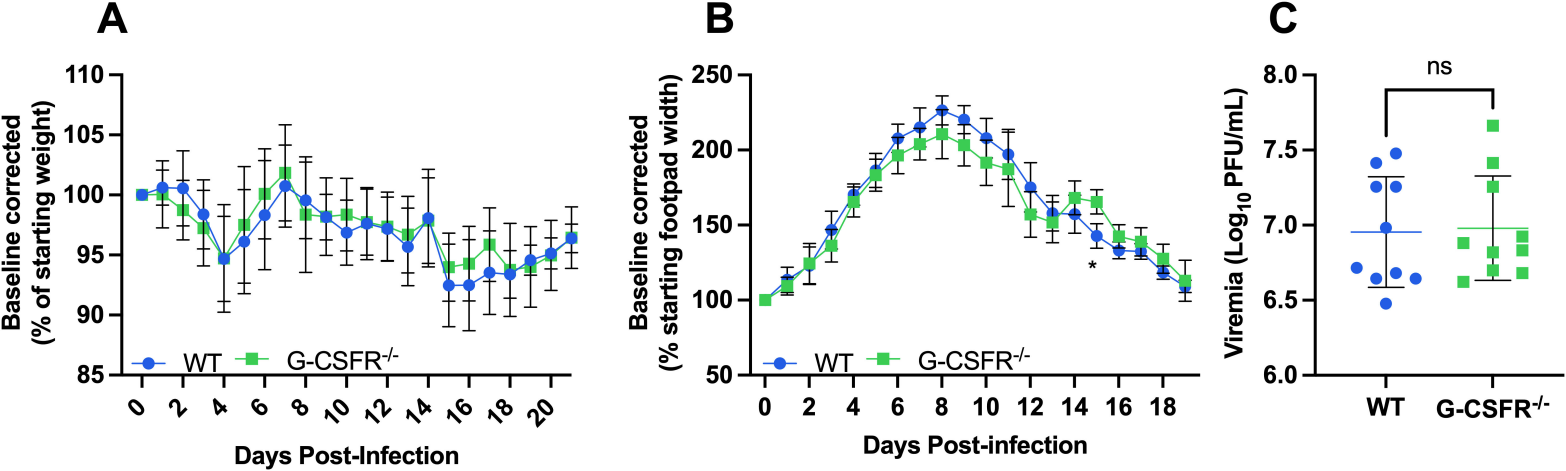
The impact of G-CSF signaling on systemic disease recovery during arthritogenic alphavirus infection depends on type I interferon signaling. C57BL/6J and G-CSFR^-/-^ mice were injected with 0.1 mg MAR1-5A3 antibody 1 day before infection to block type I IFN signaling. Mice were inoculated with 10^2^ PFU CHIKV strain SL-15649 in each hind footpad and monitored for disease development until 21 dpi (n=10). **(A-C)** Weight loss **(A)**, footpad swelling **(B)**, and viremia at 3 dpi **(C)** were measured following CHIKV infection. **(A)** Weight loss data are presented as a percentage of the starting weight of the mice on the day of inoculation. The baseline was given a value of 100%, and each mouse was tracked individually. Statistical analysis: Weight loss and footpad swelling: Mixed-effects analysis with Šídák’s multiple comparisons test. Viremia: Unpaired t-test. The error bars represent the standard deviation, bars indicate mean values, and asterisks indicate statistical differences. The level of significance represented is as follows: *p < 0.05; ns, not significant (p < 0.05). Experiments were performed in two independent biological replicates with n = 10.

Alphaviruses are highly sensitive to type I IFN restriction, and mice deficient in IFNAR-signaling are highly susceptible to disease (20, 40). Monocytes are increased in G-CSFR^-/-^ mice (**Figure 3**) following CHIKV and MAYV infection and are known to produce type I IFNs in response to CHIKV or RRV infection (31, 32). Thus, we hypothesized that type I IFN might be responsible for the worse systemic disease in G-CSFR^-/-^ mice, and that blockade of type I IFN signaling might reverse disease outcomes in these mice. To test this, we injected MAR1-5A3 antibody (39) to block IFNAR signaling one day prior to CHIKV infection. We observed a similar pattern of weight loss in CHIKV-infected WT and G-CSFR^-/-^ groups, indicating that worse systemic disease observed in G-CSFR^-/-^ mice is mediated by type I IFN signaling (**Figure 4A**). We observed only minimal differences in footpad swelling (**Figure 4B**) and no difference in viremia at 3 dpi (**Figure 4C**) between the groups. These data suggest that G-CSF signaling contributes to the control of systemic arthritogenic alphavirus disease through type I IFN signaling.

## Discussion

Arthritogenic alphavirus infection causes acute and chronic disease characterized by fever, skin rash, myalgia, and debilitating joint pain. In the infected vertebrate host, inflammatory responses are activated in target tissues such as muscles and joints, leading to the up-regulation of several proinflammatory cytokines, including G-CSF (25–28). Here, we explored G-CSF’s role in arthritogenic alphavirus disease. We infected G-CSFR-deficient mice and observed that CHIKV and MAYV infection cause sustained weight loss compared to WT mice. Blocking type I IFN signaling in G-CSFR^-/-^ mice by injecting anti-IFNAR1 antibodies reversed this phenotype. Furthermore, we observed a higher percentage of monocytes and B cells in CHIKV- and MAYV-infected G-CSFR^-/-^ mice compared to WT animals. Together, these data suggest that G-CSFR-signaling controls systemic arthritogenic alphavirus disease through type I IFN signaling.

G-CSF is elevated systemically during both acute and chronic arthritogenic alphavirus infections in humans (25–28) and mice (29). Consistent with previous reports, we found that CHIKV and MAYV infection resulted in increased G-CSF levels compared to mock-infected controls at 2 and 7 dpi (**Figure 1**). However, the kinetics differed between the two viruses: G-CSF levels declined between 2 and 7 dpi during CHIKV infection, whereas they continued to rise during MAYV infection. This difference may reflect underlying biological differences, as MAYV replicates to substantially higher systemic titers than CHIKV (**Figure 2**), potentially leading to prolonged immune activation and G-CSF secretion. Alternatively, this may be due to differences in how the data were collected: G-CSF levels in MAYV-infected mice were measured using a multiplex Luminex assay at 2 dpi, while values at 7 dpi were measured by ELISA; both 2 and 7 dpi G-CSF levels were measured using ELISA in CHIKV-infected mice. Thus, data generated for MAYV may not be comparable between 2 and 7 dpi. Importantly, this does not alter the conclusion that infection with MAYV or CHIKV leads to increased levels of systemic G-CSF.

Given the upregulation of G-CSF during infection, we hypothesized that G-CSF signaling plays a role in controlling disease severity. To test this, we inoculated G-CSFR-/- mice with MAYV and CHIKV and monitored disease progression, viral replication, and immune cell dynamics. G-CSFR-/- mice exhibited sustained weight loss compared to WT mice after infection with either virus. Notably, differences in weight trajectories emerged earlier during CHIKV infection but were delayed until 7 dpi during MAYV infection, which may correspond to the distinct G-CSF dynamics observed between the two infections. Alternatively, differences may have been mediated by differences in immune cell populations in the blood since differences in neutrophils, and anti- and pro-inflammatory monocytes appeared in 2 dpi in MAYV-infected mice but only emerged at 7 dpi in CHIKV-infected mice. In contrast, no differences were observed in footpad swelling or systemic virus replication between G-CSFR^-/-^ and WT mice. As expected, we observed that G-CSFR^-/-^ mice had a significant reduction in the proportion of blood neutrophils compared to WT mice during CHIKV and MAYV infection (22, 23). Neutrophil infiltration has been implicated in more severe footpad swelling following CHIKV infection (30); however, depletion of neutrophils in WT mice had no impact on disease outcomes (24). Neutrophils were replaced in G-CSFR^-/-^ mice with both anti- (Ly6C^lo^) and pro-inflammatory (Ly6C^hi^) monocytes, and, to a lesser extent, B cells. Recently, it has been reported that Ly6C^+^ monocytes facilitate alphavirus infection at the initial infection site, promoting more rapid viral spread into circulation (41). Furthermore, Ly6C^hi^ monocyte recruitment to the draining lymph nodes during CHIKV infection impairs virus-specific B cell responses through the production of nitric oxide (24). The increase in monocytes and B cells in the absence of G-CSFR may have led to a dysregulated adaptive immune response, possibly contributing to the sustained weight loss observed in G-CSFR^-/-^ mice. In addition, G-CSF-stimulated neutrophils may serve to control monocyte activation in the blood during arthritogenic alphavirus infection, as neutrophil-induced immune modulation has been reported previously (42–44). These data suggest that G-CSF signaling maintains a balance between neutrophil and monocyte populations during alphavirus infection, potentially regulating the activity of other immune cells. Further investigation is required to explore the complex effects of G-CSF on the functionality of neutrophils and other immune cells during alphavirus infection.

Next, we aimed to explore the mechanism by which G-CSF contributes to controlling systemic arthritogenic alphavirus disease. In previous studies, it has been reported that dysregulated type I IFNs can lead to poor disease recovery from virus infection (45–48). Therefore, we hypothesized that G-CSF signaling might contribute to the regulation of type I IFN responses in alphavirus-infected mice, mediating the control of systemic disease. When we blocked type I IFN signaling, we observed a similar pattern of weight loss in CHIKV-infected WT and G-CSFR^-/-^ mice. Notably, CHIKV has evolved mechanisms to antagonize type I IFN production and signaling (42–51). This viral immune evasion likely influences the regulation of G-CSF and neutrophil responses during infection. The observed dependence of G-CSF-driven protection on intact IFNAR signaling in our study underscores the importance of this axis in coordinating early antiviral responses and may explain variability in disease outcomes across alphaviruses. These findings suggest that G-CSF signaling may play a protective role in WT mice by modulating type I IFN signaling. Future studies should investigate the distinct roles of IFN-α and IFN-β in G-CSF’s modulation of immune cell dynamics, as these type I IFNs have been shown to play distinct roles in controlling CHIKV replication and neutrophil infiltration (20, 40).

In summary, our study sheds light on the role of G-CSF signaling in the pathogenesis of CHIKV and MAYV. Our findings demonstrate that G-CSF signaling plays a crucial role in controlling systemic disease during alphavirus infection. G-CSFR deficiency resulted in a marked reduction in neutrophilsin the blood during infection, which were replaced by anti-inflammatory and pro-inflammatory monocytes and B cells. Additionally, the effect of G-CSFR deficiency on weight loss was found to be dependent on type I interferon signaling. Given these insights, further exploration of G-CSF’s role at the molecular level in arthritogenic alphavirus disease is warranted. This could pave the way for its potential development as a therapeutic target for both acute and chronic forms of the disease.

## Data Availability

The original contributions presented in the study are included in the article/**Supplementary Material**. Further inquiries can be directed to the corresponding author.
