# Supplementary material for: Granulocyte colony-stimulating factor protects against acute systemic alphavirus disease in a type I IFN-dependent manner
Source: Front Immunol. 2025 Jul 11;16:1606053. doi: 10.3389/fimmu.2025.1606053 (PMC12289501; doi:10.3389/fimmu.2025.1606053)
Supplement: Supplementary file 1 [file Supplementaryfile1.docx]

**Supplementary Figure 1.** Heterozygous colony-stimulating factor 3 receptor gene (Csf3r or G-CSFR) knockout mice (B6.129X1 (Cg)-Csf3r^tm1Link/J^) were purchased from The Jackson Laboratory (JAX) at 6-8 weeks of age. Heterozygous G-CSFR^+/-^ mice were bred to obtain homozygous G-CSFR^-/-^ mice. Homozygous G-CSFR^-/-^ mice were used in all studies. Genotyping was performed as recommended by JAX. Briefly, DNA was isolated from tail snips from pups and the following primers were used for amplification: Common Primer Forward (ACATAAGCCTGTGGGAAGG), Wild-Type (WT) Reverse (GCTGGTTCTCCACTCATTTG), and Mutant Reverse (CTCCAGACTGCCTTGGGAAAA). Expected band sizes are 78 bp for WT, 136 bp for G-CSFR^-/-^, and both bands for heterozygotes.

**
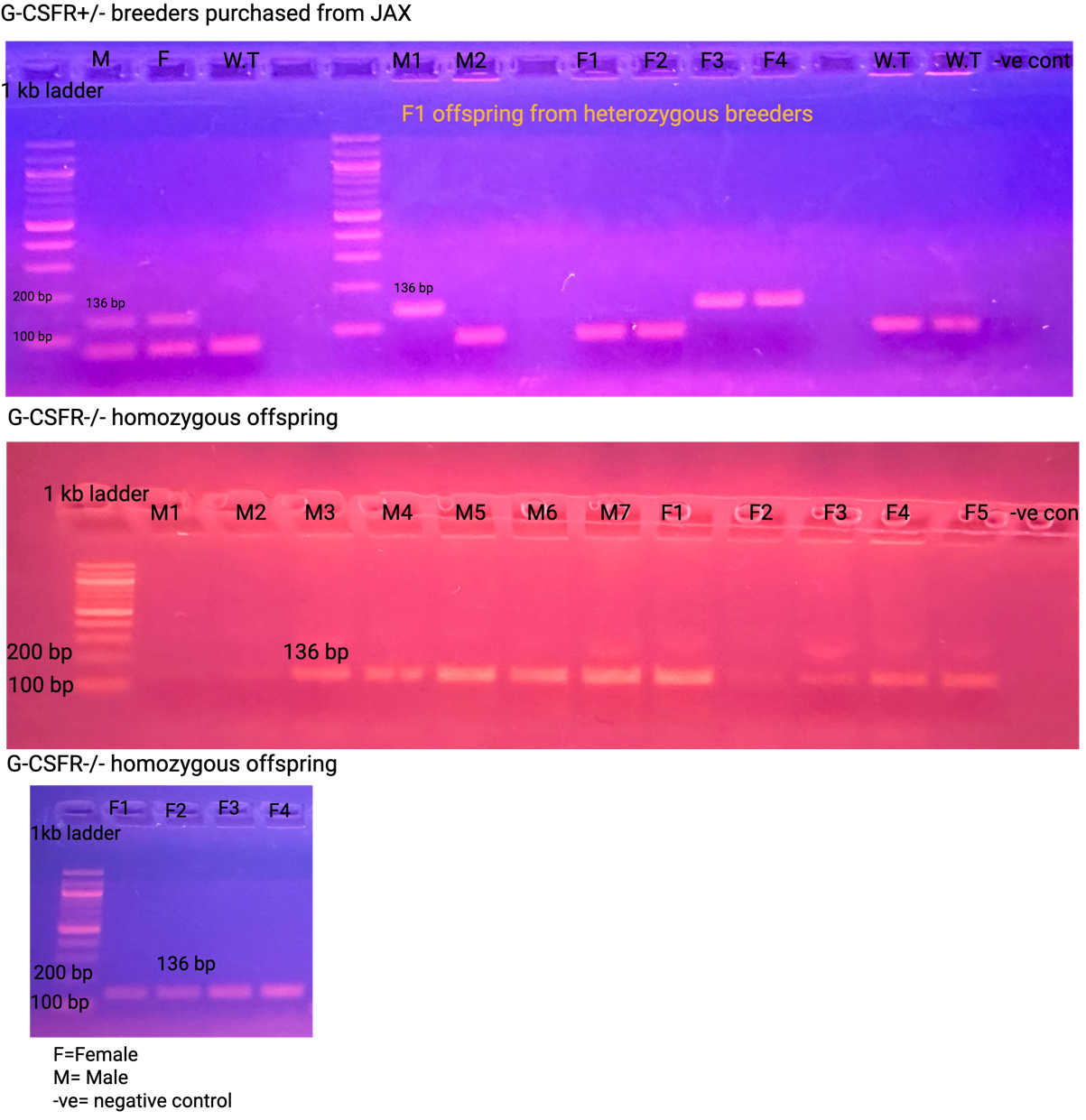
**
