# Supplementary material for: Granulocyte colony-stimulating factor protects against acute systemic alphavirus disease in a type I IFN-dependent manner
Source: Front Immunol. 2025 Jul 11;16:1606053. doi: 10.3389/fimmu.2025.1606053 (PMC12289501; doi:10.3389/fimmu.2025.1606053)
Supplement: Supplementary file 2 [file Supplementaryfile2.docx]

**Supplementary Figure 2.** Gating strategy for flow cytometry**.**
