# Supplementary material for: Granulocyte colony-stimulating factor protects against acute systemic alphavirus disease in a type I IFN-dependent manner
Source: Front Immunol. 2025 Jul 11;16:1606053. doi: 10.3389/fimmu.2025.1606053 (PMC12289501; doi:10.3389/fimmu.2025.1606053)
Supplement: Supplementary file 3 [file Supplementaryfile3.docx]

**Supplementary Figure 3. G-CSF deficiency leads to a systemic increase in B cells during arthritogenic alphavirus infection.**

C57BL/6J and G-CSFR^-/-^ mice were inoculated with 10^4^ PFU of MAYV strain TRVL 4675 (n=5) or 10^5^ PFU of CHIKV strain SL-15649 (n=4) in each hind footpad, and blood was collected at the indicated days post-infection (DPI). Leukocytes were isolated from the blood and subjected to flow cytometry. **A-F.** Dot plots presenting the percentage of CD4 T cells (CD45^+^CD3^+^CD4^+^) (**A and D**), CD8 T cells (CD45^+^CD3^+^CD8^+^) (**B and E**), and B cells (CD45^+^CD3^-^CD19^+^) (**C and F**) in WT and G-CSFR^-/-^ groups during MAYV and CHIKV infection. Statistical analysis: 2way ANOVA with Šídák's multiple comparisons test. The error bars represent the standard deviation, bars indicate mean values, and asterisks indicate statistical differences. The level of significance represented is as follows: *p < 0.05, **p < 0.01.

**
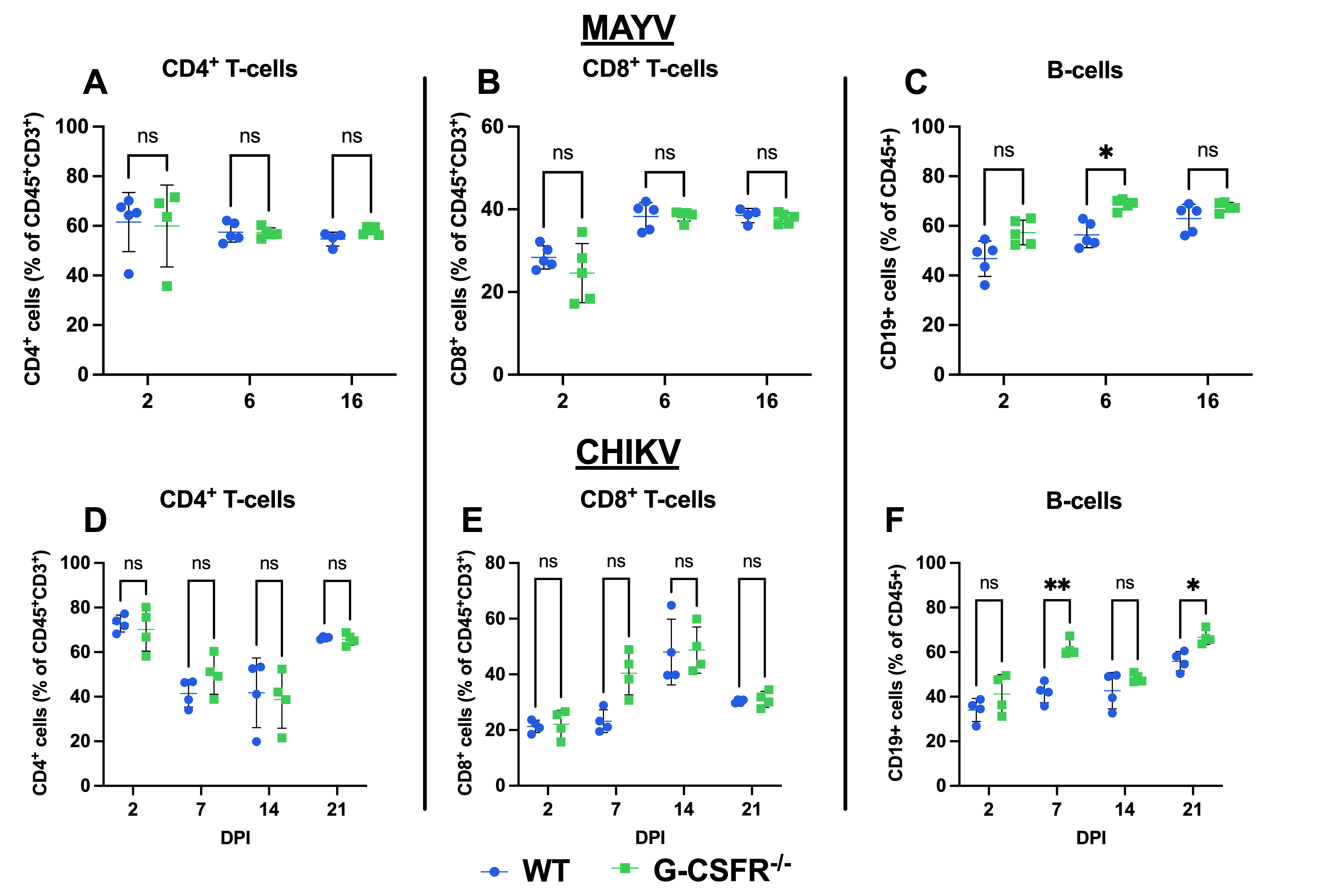
**
