# Supplementary material for: Granulocyte colony-stimulating factor protects against acute systemic alphavirus disease in a type I IFN-dependent manner
Source: Front Immunol. 2025 Jul 11;16:1606053. doi: 10.3389/fimmu.2025.1606053 (PMC12289501; doi:10.3389/fimmu.2025.1606053)
Supplement: Supplementary file 4 [file Supplementaryfile4.docx]

**Supplementary Figure 4. G-CSF deficiency does not alter early virus-specific antibody responses during arthritogenic alphavirus infection.**

C57BL/6J and G-CSFR^-/-^ mice were inoculated with 10^4^ PFU of MAYV strain TRVL 4675 (n=4) in each hind footpad, and blood was collected at 13 days post-infection (DPI). A plaque-reduction neutralization test using an 80% threshold (PRNT_80_) was used to assess virus-specific neutralizing antibodies. Statistical analysis: unpaired t-test. The error bars represent the standard deviation, bars indicate mean values, and asterisks indicate statistical differences. Results were repeated twice with similar findings.

**
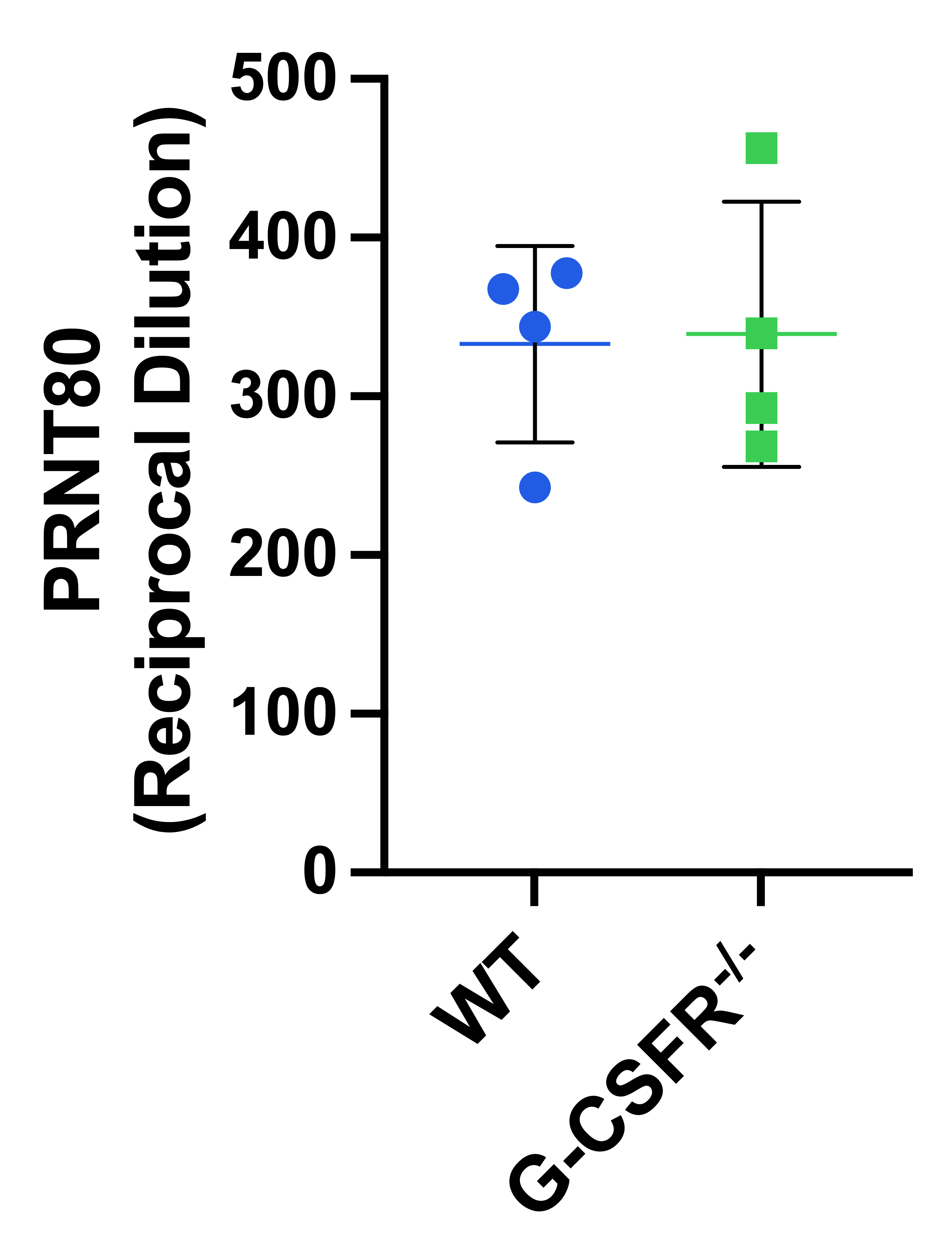
**
