# Supplementary material for: Granulocyte colony-stimulating factor protects against acute systemic alphavirus disease in a type I IFN-dependent manner
Source: Front Immunol. 2025 Jul 11;16:1606053. doi: 10.3389/fimmu.2025.1606053 (PMC12289501; doi:10.3389/fimmu.2025.1606053)
Supplement: Supplementary file 5 [file Supplementaryfile5.docx]

**Supplementary Figure 5. G-CSF deficiency does not alter serum levels of IFNγ and TNFα at 6 days post-MAYV infection.**

C57BL/6J and G-CSFR^-/-^ mice were inoculated with 10^4^ PFU of MAYV strain TRVL 4675 (n=5) in each hind footpad, and blood was collected at 6 days post-infection (DPI). ELISA was used to measure serum levels of IFNγ and TNFα. Statistical analysis: unpaired t-test. The error bars represent the standard deviation, bars indicate mean values, and values indicate p-values.

**
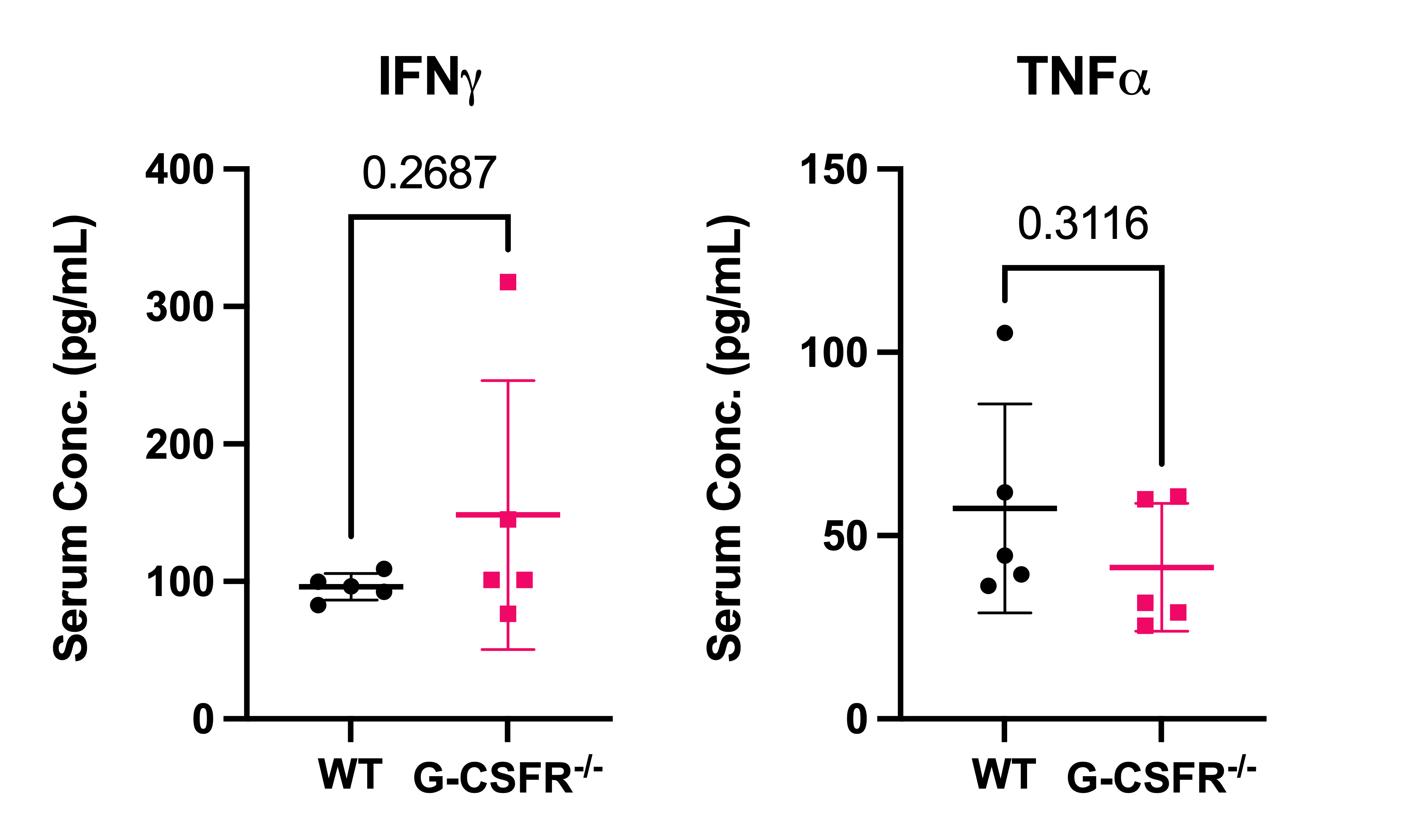
**
